# Highly Pathogenic Avian Influenza (H5N1) Outbreaks in Wild Birds and Poultry, South Korea

**DOI:** 10.3201/eid1803.111490

**Published:** 2012-03

**Authors:** Hye-Ryoung Kim, Youn-Jeong Lee, Choi-Kyu Park, Jae-Ku Oem, O-Soo Lee, Hyun-Mi Kang, Jun-Gu Choi, You-Chan Bae

**Affiliations:** Animal, Plant and Fisheries Quarantine and Inspection Agency, Anyang, South Korea

**Keywords:** 2.3.2 clade, highly pathogenic avian influenza, HPAI, H5N1, influenza A virus, South Korea, influenza, viruses, respiratory infections, birds, poultry

## Abstract

Highly pathogenic avian influenza (H5N1) among wild birds emerged simultaneously with outbreaks in domestic poultry in South Korea during November 2010–May 2011. Phylogenetic analysis showed that these viruses belonged to clade 2.3.2, as did viruses found in Mongolia, the People’s Republic of China, and Russia in 2009 and 2010.

Since 2003, highly pathogenic influenza (HPAI) virus subtype H5N1 has become enzootic in some countries and continues to cause outbreaks in poultry and sporadic cases of infection in humans, thus posing a persistent potential pandemic threat (*1*). Wild birds, especially waterfowl of the order Anseriformes (ducks, geese, and swans) are the natural reservoir of low pathogenicity avian influenza viruses, and since the HPAI outbreak at Lake Qinghai, People’s Republic of China, in 2005, they have been suspected of playing a role as long-distance vectors of HPAI viruses (*2**,**3*).

Three previous outbreaks in South Korea are assumed, on the basis of epidemiologic evidence, to have been caused by HPAI (H5N1) viruses introduced by migratory birds, although a carcass or moribund wild bird infected with these viruses (which would serve as a link to the introduction of infection in domestic poultry) was not found (*4**–**6*). On December 7, 2010, an HPAI (H5N1) virus was isolated from a healthy mallard in South Korea (*7*). After that, subtype H5N1 viruses were frequently detected in wild birds and poultry until May 2011. In this study, we analyzed the epidemiologic features of this outbreak and investigated the characteristics of strains through genetic analysis.

## The Study

**Figure 1:**
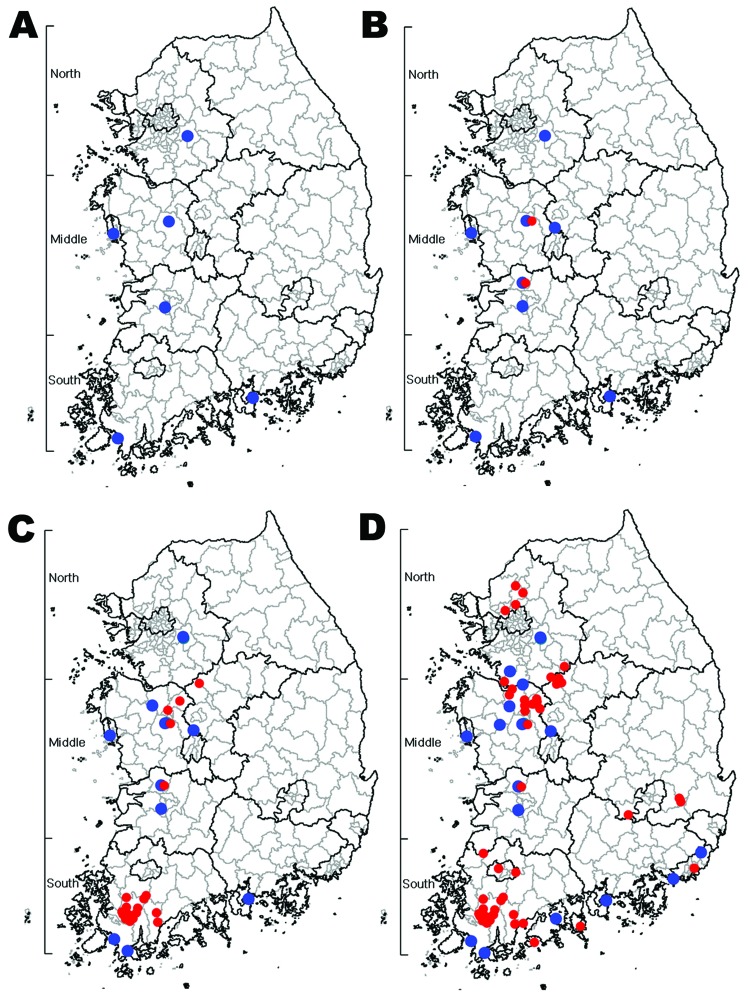
Progress of highly pathogenic avian influenza (HPAI) outbreak by time, South Korea, 2010–2011. A) HPAI-positive cases identified from samples collected November 26–December 28, 2010 (wild birds, 6 cases). B) Cases identified by January 4, 2011 (wild birds, 10 cases; poultry, 2 cases). C) Cases identified by January 11, 2011 (wild birds, 13 cases; poultry, 23 cases). D) Cases identified by May 16, 2011 (wild birds, 20 cases; poultry, 53 cases). Blue circles indicate locations where HPAI viruses were isolated from wild birds; red circles indicate locations where HPAI viruses were isolated from poultry**.**

From November 26 to December 28, 2010, 6 cases of subtype H5N1 infection were identified in carcasses, feces, and cloacal swab specimens of migratory birds collected throughout South Korea. Subtype H5N1 viruses were found in various bird species (mallard, Baikal teal, mandarin duck, whooper swan, and Eurasian eagle owl) in places such as migratory bird habitats and nearby hills (Figure 1, panel A). Despite the repeated predictions from animal health authorities of poultry outbreaks and the emphasis on protection against contamination, on December 30, 2010, HPAI was confirmed on 2 poultry farms. These farms are located in the middle region of South Korea and are close (distances of 1.3 km and 0.4 km, respectively) to the migratory habitats of birds that have been positive for subtype H5N1 virus (Figure 1, panel B). During the next 2 weeks, the percentage of HPAI-positive birds increased rapidly among clustered poultry farms located in the southern region, and poultry on 23 farms and 13 wild birds were confirmed to be infected with HPAI virus (Figure 1, panel C). The HPAI outbreak spread more slowly until May 16, 2011, and an additional 30 poultry farms and 7 wild birds were confirmed to be infected with subtype H5N1 virus (Figure 1, panel D).

Fourteen bird species were found to be positive for subtype H5N1 (Table). The affected poultry included species of the order Galliformes (chickens, quail, etc.), which exhibited sudden death with severe clinical signs, and domestic ducks (order Anseriformes), which died suddenly or exhibited a decrease in egg production, depending on age. Most infected birds of species that belonged to the orders Anseriformes, Falconiformes, and Strigiformes were found dead, but a few infections were detected in swab specimens from healthy mallards and feces of wild birds. Moreover, species of Anseriformes, such as the Mandarin duck, were dominant among the wild birds with HPAI until the beginning of January 2011. After that time, many HPAI infections were found in birds of prey, such as the Eurasian eagle owl (Table).

All viruses were isolated by inoculating embryonated chicken eggs with specimens from cloacal swab specimens, feces, and homogenized organs from birds with suspected infections. The hemagglutinin (HA) and neuraminidase (NA) proteins were subtyped as previously described (*6*). We selected 27 viruses, taking into consideration the outbreak period, the region, and the host species (Table A1) and conducted sequencing and phylogenetic analysis of 8 gene segments. The genome sequences of 27 viruses are available from GenBank under accession numbers JN807892–JN808107.

**Figure 2:**
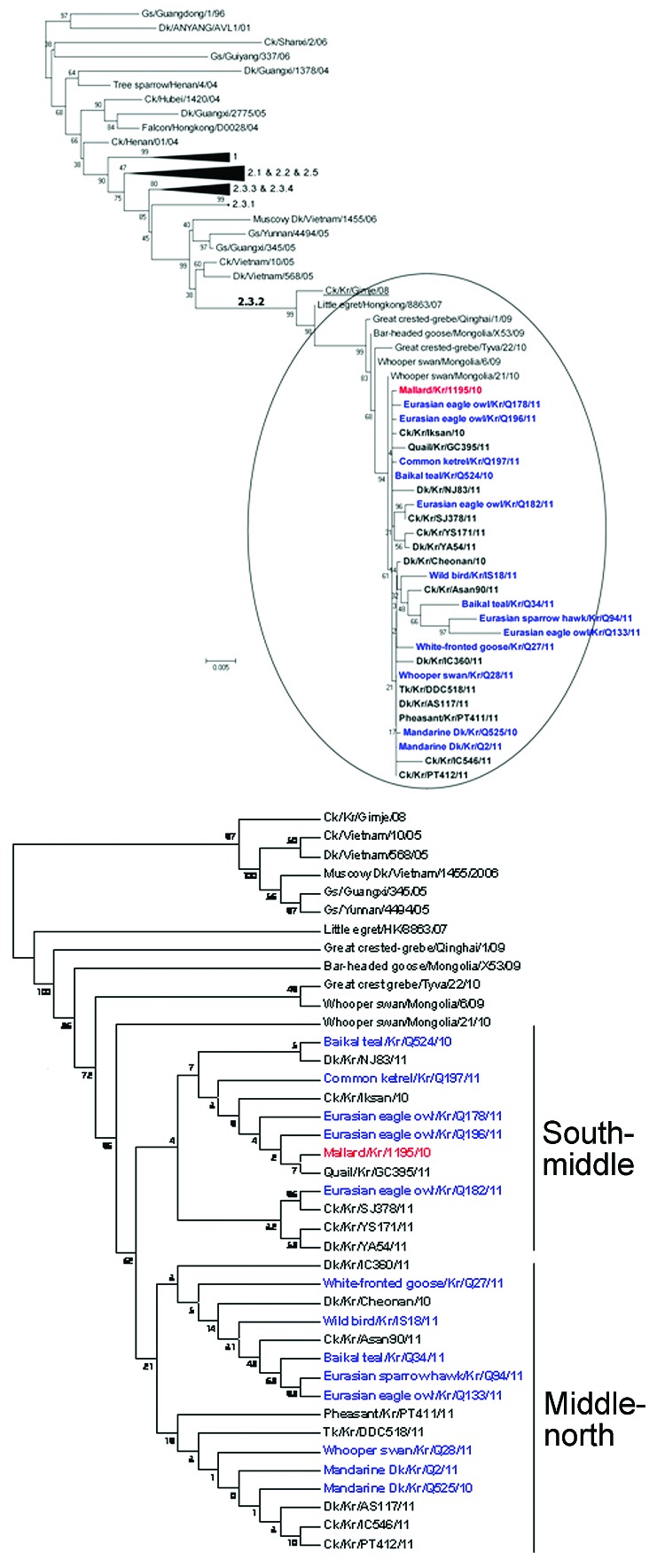
Phylogenetic diagram of hemagglutinin (HA) gene of highly pathogenic avian influenza (H5N1) viruses, including viruses isolated in South Korea during 2010–2011. Blue indicates viruses isolated from wild birds, boldface indicates isolates from poultry, and red indicates reference virus.

In the HA phylogenetic tree, all 27 viruses were clustered into clade 2.3.2 HPAI viruses, together with the subtype H5N1 virus that had been isolated from a healthy mallard (*7*). All isolates showed a high HA homology (>99.5%) (Table A1). Of note, the isolates from poultry fell into 2 sublineages, south-middle and north-middle, which were distinct geographic regions in the HPAI outbreak among poultry, but the isolates from wild birds were not subgrouped in the phylogenetic tree, which displays only topology (Figure 2).

**Figure A1:**
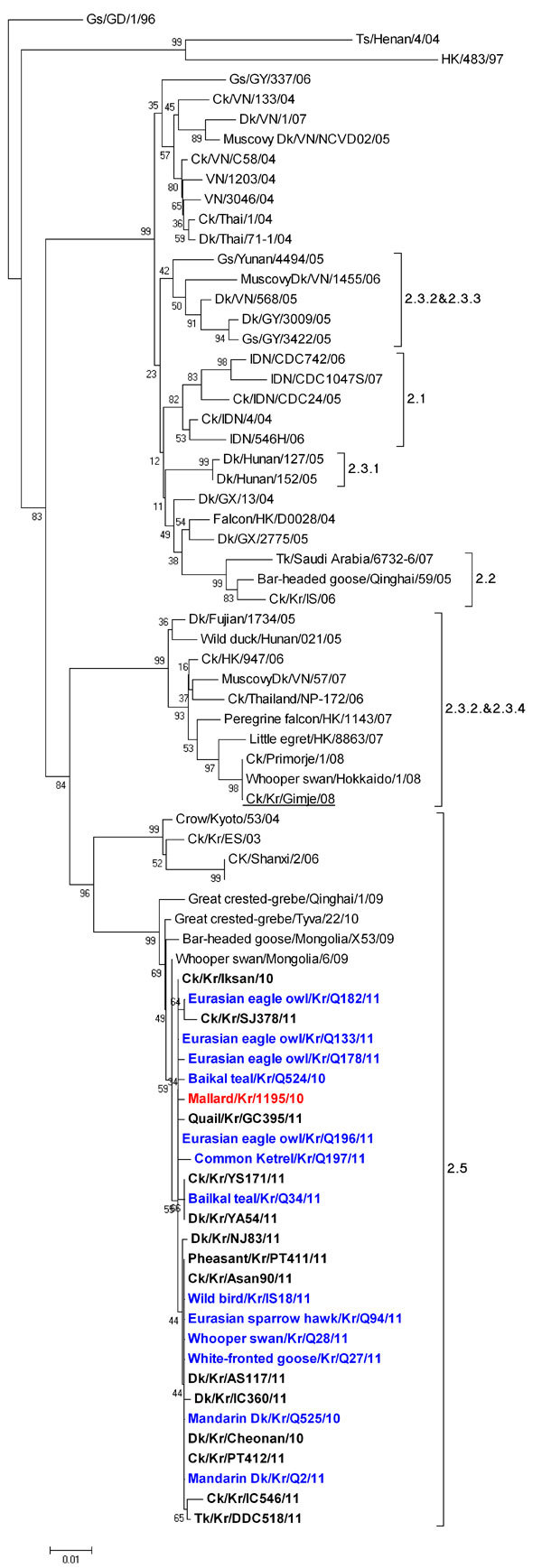
Phylogenetic diagram of acidic polymerase gene of highly pathogenic avian influenza (H5N1) viruses, including viruses isolated in South Korea during 2010–2011. Blue indicates viruses isolated from wild birds, boldface indicates isolates from poultry, and red indicates reference virus.

These isolates were different from the HPAI viruses responsible for previous outbreaks in South Korea (A/chicken/Korea/ES/2003[clade 2.5], A/chicken/Korea/IS/2006[clade 2.2] and A/chicken/Korea/Gimje/2008[clade 2.3.2]) and were closely related (>99%) to the subtype H5N1 isolates found in Mongolia, China, and Russia in 2009–2010. The phylogenetic analysis also showed that the NA and other internal genes were closely related to those of subtype H5N1 viruses found in wild birds in Mongolia, China, and Russia in 2009–2010 (Figure A1; unpub. data).

All 27 viruses characterized were highly pathogenic and had variations in the multibasic cleavage site in the HA molecule (PQRERRRKR) and a 20-aa deletion in the stalk region of NA. They did not have amino acid substitutions that conferred resistance to amantadine or oseltamivir and were associated with the increased virulence of subtype H5N1 viruses in mammalian hosts (*8*).

The intravenous pathogenicity test was conducted by using the A/duck/Korea/Cheonan/2010 virus, the first isolate from poultry. The intravenous pathogenicity index was 3.0 for chickens.

## Conclusion

After the outbreak at Lake Qinghai, China, in 2005, the clade 2.2 viruses spread from Asia to Europe and Africa during 2005–2006 and have been circulating widely in southern Asia, the Middle East, Europe, and Africa for several years. Clade 2.3.2 viruses might spread over an extensive area, similar to clade 2.2 viruses, because clade 2.3.2 viruses are widespread among wild birds and have been continuously evolving in the regions where subtype H5N1 viruses are endemic (*9*). Clade 2.3.2 viruses have circulated in Vietnam and southern China since 2005, and clade 2.3.2 viruses that had undergone reassortment with clade 2.3.4 viruses were isolated from wild birds in Hong Kong in 2007. These reassorted viruses caused HPAI (H5N1) outbreaks in Japan, Russia, and South Korea during 2008 (*6**,**10**–**12*). New 2.3.2 viruses, reassortants that possessed a different acidic polymerase gene from the 2.3.2 viruses of 2007–2008, were isolated predominantly from migratory birds in Mongolia and China in 2009–2010 (*13**,**14*). No HPAI outbreaks occurred in South Korea and Japan in 2009, but outbreaks of a similar virus took place in both countries in late 2010 (*15*). The situation was analogous to outbreaks in 2 countries in 2006–2007 by clade 2.2 HPAI viruses that had been detected in China, Mongolia, and Russia in 2005. Thus, the migratory patterns of infected wild birds might be related to these outbreaks.

During the initial stage of the 2010–2011 outbreak, HPAI viruses were detected in several wild birds, and the viruses were assumed to have been introduced into domestic poultry by migratory birds. The detection of HPAI (H5N1) virus in free-ranging migratory bird might predict a poultry outbreak if biosecurity measures in poultry are inadequate, but during 2006, several European countries reported HPAI (H5N1) virus infections in wild birds without concurrent poultry outbreaks. In the 2010-2011 outbreak in South Korea, the subsequent outbreak cases suggest that the subtype H5N1 virus was spread from farm to farm by humans and associated agricultural practices so that strains of poultry were grouped in sublineages by region.

Clade 2.3.2 subtype H5N1 viruses have been circulating in poultry and migratory birds in Asia and have accumulated antigenic mutations. We can conclude that early detection of HPAI outbreaks and a rapid response to them are essential in controlling the introduction of virus from migratory birds to poultry and in preventing farm-to-farm spread.

## Appendix

**Table A1:** Details of tested subtype H5N1 avian influenza viruses from wild birds and poultry, South Korea, 2010–2011*

Isolate	Region	Date isolated	HA1 gene homology, %	Sample (breed)
Wild birds				
Mallard/Korea/1195/2010†	Middle	2010 Dec 7	–	Cloacal swab
Baikal teal/Korea/Q524/2010	South	2010 Dec 27	99.9	TCK
Mandarin duck/Korea/Q525/2010	South	2010 Dec 31	99.8	TCK
Wild bird/Korea/IS18/2011	Middle	2011 Jan 5	99.8	Feces
Mandarin duck/Korea/Q2/2011	South	2011 Jan 5	99.8	TCK
Whooper swan/Korea/Q28/2011	North	2011 Jan 10	99.7	TCK
Baikal teal/Korea/Q34/2011	South	2011 Jan 10	99.6	TCK
White-fronted goose/Korea/Q27/2011	Middle	2011 Jan 10	99.7	TCK
Eurasian sparrowhawk/Korea/Q94/2011	North	2011 Jan 21	99.5	TCK
Eurasian eagle owl/Korea/Q133/2011	Middle	2011 Jan 27	99.5	TCK
Eurasian eagle owl/Korea/Q178/2011	Middle	2011 Feb 3	99.7	TCK
Eurasian eagle owl/Korea/Q182/2011	North	2011 Feb 4	99.5	TCK
Eurasian eagle owl/Korea/Q196/2011	South	2011 Feb 10	99.7	TCK
Common kestrel/Korea/Q197/2011	South	2011 Feb 11	99.8	TCK
Poultry				
Duck/Korea/Cheonan/2010	Middle	2010 Dec 30	99.8	TCK (B)
Chicken/Korea/Iksan/2010	Middle	2010 Dec 30	99.9	TCK (bB)
Duck/Korea/YA54/2011	South	2011 Jan 7	99.8	TCK (b)
Duck/Korea/NJ83/2011	South	2011 Jan 9	99.8	TCK (B)
Chicken/Korea/Asan90/2011	Middle	2011 Jan 8	99.7	TCK (L)
Duck/Korea/AS117/2011	North	2011 Jan 10	99.7	TCK (b)
Chicken/Korea/YS171/2011	South	2011 Jan 14	99.6	TCK (knc)
Duck/Korea/IC360/2011	North	2011 Jan 23	99.8	feces (b)
Chicken/Korea/SJ378/2011	Middle	2011 Jan 24	99.6	TCK (L)
Quail/Korea/GC395/2011	Middle	2011 Jan 25	99.8	TCK (L)
Pheasant/Korea/PT411/2011	North	2011 Jan 27	99.8	TCK (b)
Chicken/Korea/PT412/2011	North	2011 Jan 27	99.8	TCK (bB)
Turkey/Korea/DDC518/2011	North	2011 Feb 13	99.7	TCK (b)
Chicken/Korea/IC546/2011	North	2011 Feb 18	99.7	TCK (bB)

*HA, hemagglutinin; TCK, trachea, cecal tonsil, kidney; B, breeder; bB,broiler breeder; b, broiler; L, layer; knc, Korean native chicken. †Source (*7*).

**Table:** Bird species that tested positive for highly pathogenic avian influenza subtype H5N1 in South Korea, 2010–2011

Avian order and species	Scientific name	Sample	No. positive
Galliformes*			
Domestic chicken	*Gallus gallus domesticus*	Carcasses, feces	18
Common quail	*Coturnix coturnix*	Carcasses, feces	1
Common pheasant	*Phasianus colchicus*	Carcasses, feces	1
Domestic turkey	*Meleagris gallopavo*	Carcasses, feces	1
Anseriformes			
Domestic duck*	*Anas platyrhynchos*	Carcasses, feces	32
Mallard	*Anas platyrhynchos*	Cloacal swab specimen, † feces	2
Mandarin duck	*Aix galericulata*	Carcasses, feces	5
Whooper swan	*Cygnus cygnus*	Carcass	1
Baikal teal	*Anas formosa*	Carcasses	2
White-fronted goose	*Anser albifrons*	Carcass	1
Spot-billed duck	*Anas poecilorhyncha*	Carcass	1
Falconiformes			
Eurasian sparrowhawk	*Accipiter nisus*	Carcass	1
Common kestrel	*Falco tinnunculus*	Carcass	1
Strigiformes			
Eurasian eagle owl	*Bubo bubo*	Carcasses	5
Unknown	–	Feces	1
Total			73

*Galliformes and domestic ducks were from poultry farms. †Source (*7*).
